# Quantitative Image Analysis and Modeling Indicate the *Agrobacterium tumefaciens* Type IV Secretion System Is Organized in a Periodic Pattern of Foci

**DOI:** 10.1371/journal.pone.0042219

**Published:** 2012-07-30

**Authors:** Todd A. Cameron, Marcus Roper, Patricia C. Zambryski

**Affiliations:** 1 Department of Plant and Microbial Biology, University of California, Berkeley, California, United States of America; 2 Department of Mathematics, University of California Los Angeles, Los Angeles, California, United States of America; Institut Pasteur Paris, France

## Abstract

The Gram negative plant pathogen *Agrobacterium tumefaciens* is uniquely capable of genetically transforming eukaryotic host cells during the infection process. DNA and protein substrates are transferred into plant cells via a type IV secretion system (T4SS), which forms large cell-envelope spanning complexes at multiple sites around the bacterial circumference. To gain a detailed understanding of T4SS positioning, the spatial distribution of fluorescently labeled T4SS components was quantitatively assessed to distinguish between random and structured localization processes. Through deconvolution microscopy followed by Fourier analysis and modeling, T4SS foci were found to localize in a non-random periodic pattern. These results indicate that T4SS complexes are dependent on an underlying scaffold or assembly process to obtain an organized distribution suitable for effective delivery of substrates into host cells.

## Introduction

The type IV secretion system (T4SS) is an evolutionarily conserved bacterial secretion apparatus that is essential for conjugation and effector protein secretion during numerous pathogenic interactions between bacteria and their eukaryotic hosts (for review, see ref [1]). Many notable human pathogens, including *Brucella ssp.*, *Bordetella ssp.*, *Legionella ssp.* and *Coxiella burnetii* rely on T4SSs for effective host colonization [2], [3]. However, one of the first recognized and best characterized T4SS is that of the soil-dwelling plant pathogen *Agrobacterium tumefaciens*. The T4SS of *A. tumefaciens* serves as a general model for T4SS structure and function.

*A. tumefaciens* cells undergo virulence (*vir*) induction when stimulated simultaneously by several signals, including lowered pH, certain sugars, and phenolic compounds such as acetosyringone released from wounded plant tissues [4]–[7]. In *vir*-induced cells, T4SS genes in the *virB* operon are expressed to produce *virB* T4SS complexes. Eleven proteins, VirB1 through VirB11, form a large cell envelope-spanning T4SS complex and extracellular T-pilus that together mediate the delivery of T4SS substrates [8]–[11]. Recent structural analyses have established that fourteen copies of VirB7, VirB9 and VirB10 together form a large core complex approximately 20 nm in diameter and more than 1 mDa in size [12], [13]. The complex is further composed of multiple copies of the ATPases VirB4 and VirB11, additional proteins of structural or functional significance (VirB1, VirB3, VirB6, VirB8), and the major and minor T-pilus components VirB2 and VirB5.

The *virB* T4SS is uniquely capable of delivering both DNA and protein substrates into the cytoplasms of host cells. The secreted single-stranded DNA substrate, the T-strand, is directed into the plant nucleus by additional secreted chaperones (VirE2, VirF), and integrated stably into the plant genomic DNA (for review, see ref [14]). The T-strand carries bacterial genes that disrupt the balance of the plant hormones auxin and cytokinin, leading to the formation of crown gall tumors [15], [16]. Other genes carried by the T-strand promote the synthesis of opines, unusual N-carboxyalkyl amino acids that serve as carbon and nitrogen sources specifically metabolized by *A. tumefaciens* [17].

**Figure 1:**
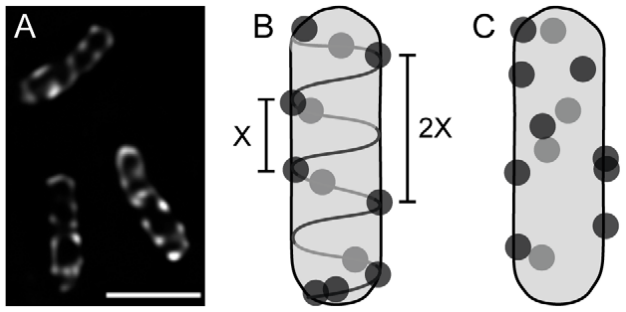
GFP-VirB8 localizes as multiple foci along the cell periphery. A, *A. tumefaciens* expressing *vir*-inducible GFP-VirB8 on plasmid pJZ041. Image represents an average intensity z-projection of a deconvolved z-stack. Scale bar is 2 µm. B and C, two possible models of the localization of GFP-VirB8. Light and dark grey circles represent foci on the far and near sides of the cell, respectively. B, helical distribution model illustrating an underlying cellular scaffold directing foci localization. The fundamental period (X) is reflected in the spacing (2X) between more distant foci pairs; gaps arise occasionally as variations in foci placement on the scaffold leads to some segments without foci. C, random distribution model of foci along cell periphery.

Recent results show that *vir* induced *A. tumefaciens* primarily attach laterally to host plant cells [18], yet non-*vir* induced bacteria attach to generic substrates (such as glass slides) using a polar holdfast [19]. This shift to a lateral attachment orientation requires *vir* induction of *A. tumefaciens*; loss of the Ti plasmid (which carries the *virB* operon encoding the T4SS and T-pili) or lack of *vir* induction both result in cells that no longer attach laterally, suggesting that the *virB* T4SS plays a role in lateral attachment [18]. To fully understand how the *virB* T4SS could participate in lateral attachment to host cells during a successful infection, it is important to determine where and how the T4SS complexes localize in the bacterial cell. Although some previous studies suggested that VirB complexes are found only at cell poles [20]–[22], improved microscopy techniques have demonstrated that *virB* T4SS component proteins and substrates localize as multiple lateral foci around the cell envelope [18], [23]. Exemplifying these results, Figure 1A and Videos S1 and S2 show that the T4SS component VirB8 localizes as apparently regularly-spaced foci along the cell periphery when fused to green fluorescent protein (GFP). This localization pattern was confirmed by detecting native VirB proteins with immunofluorescence microscopy, which avoids potential artifacts generated by overexpression of fusion proteins [18], [23]. Furthermore, since VirB8 is only stable when complexed with other VirB proteins [24], and GFP-VirB8 fully complements DNA transfer to plant cells [23], the VirB8 fusion proteins should represent only functional T4SS complexes. The lateral distribution of VirB foci strikingly parallels the lateral attachment observed of *A. tumefaciens* to host cells, suggesting that multiple lateral VirB complexes might facilitate lateral attachment and efficient substrate transfer from any side of contact.

Notably, the localization of T4SS components also resembles the multiple equally spaced foci of numerous bacterial proteins reported to localize in a helical fashion, including MreB, FtsZ, MinD, and the Sec translocase [25]–[28]. This similar pattern of localization suggests that VirB complexes might therefore be similarly organized, presumably mediated through an association with an underlying scaffold, or regularly repeating process in the cell such peptidoglycan synthesis (Fig. 1B). However, recent re-examinations of MreB localization have generally concluded that the originally proposed filamentous helical model for MreB was incorrect, and instead suggest that individual MreB patches move circumferentially around the bacterial cell [29]–[32] (see ref [33] for an alternate interpretation). Thus, it is apparent that initial appearances of spatial organization need to be thoroughly tested. For instance, the appearance of regularly spaced T4SS foci might occur by chance when observing many cells with randomly placed foci (Fig. 1C, Fig. S1).

To understand if any fundamental biological mechanisms are driving T4SS positioning, we directly tested whether VirB complexes are distributed randomly or with a regular organization. Fourier analyses, nearest neighbor distances, and modeling revealed that T4SS foci conformed to a non-random distribution with predictable periodicity. Together, the data strongly support a model where T4SS complexes are systematically spaced across the bacterial cell surface, likely to help maximize effective contact and transfer of substrates to host cells.

## Results

To determine whether GFP-VirB8 foci might occur in regular intervals, we examined the spacing of foci along the edges of *vir*-induced *A. tumefaciens* cells expressing GFP-VirB8. The analysis was conducted on foci along cell edges, since deconvolved z-stacks could be flattened to bring all of these foci into view without generating large ambiguities. As it is not possible to distinguish between foci originating from the top and bottom of an individual cell once the z-stack is flattened, we did not analyze foci in cell centers.

**Figure 2:**
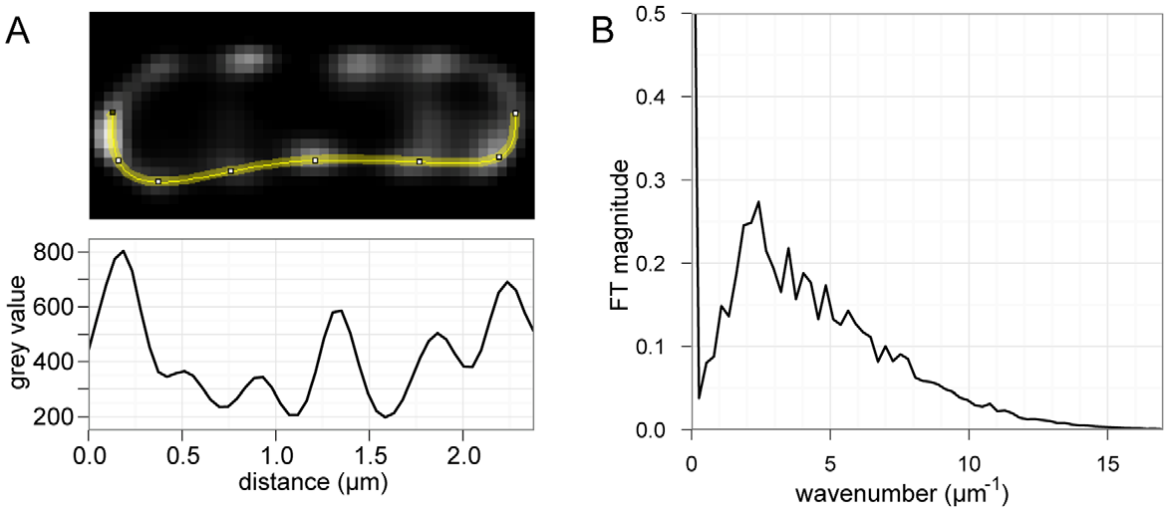
Fourier analysis reveals periodicity of GFP-VirB8 foci. A, example GFP-VirB8 expressing cell with the lower edge highlighted, with corresponding fluorescence profile plot used in subsequent analyses. B, Combined Fourier transforms of each standardized fluorescence profile from 152 cell-sides. The Fourier transform peaks between k = 1.88 and k = 2.42, indicating the presence of a periodic element with a peak period of 0.41–0.53 µm.

A Fourier analysis of the fluorescence signal along bacterial cell edges was performed to resolve variations in fluorescence intensity into component periodic signals, and reveal the presence of any predominant periodicities. Fluorescent profiles were collected along both sides of the visible cells, yielding linear intensity profiles suitable for one-dimensional Fourier analysis (Fig. 2A). A Fourier analysis decomposes a temporal or spatial signal, such as a sound or image, into component sinusoidal waves of specific frequencies and phases. The distribution of wave frequencies reflects the periodicity of the signal, with sharper peaks corresponding to the wavelengths of any periodic components of the signal. Since Fourier transformations of the raw intensity profiles resulted in periodic components describing variations in both spacing as well as foci intensity, foci peaks were standardized as Gaussian distributions of similar intensities. The resulting profiles were Fourier transformed for each cell to obtain the periodicities reflecting the spatial arrangements of foci. The cumulative periodicity of the Fourier transforms indicated that GFP-VirB8 foci were loosely periodic, with the major peak present between k = 1.88 and k = 2.41 µm^−1^ (Fig. 2B) corresponding to foci spaced about 0.41 to 0.53 µm apart. Furthermore, the smaller peaks at higher wavenumbers correspond to linear combinations of the fundamental modes k_1_ = 1.08 µm^−1^ (0.93 µm) and k_2_ = 2.41 µm^−1^ (0.41 µm), consistent with an overall periodic spacing.

**Figure 3:**
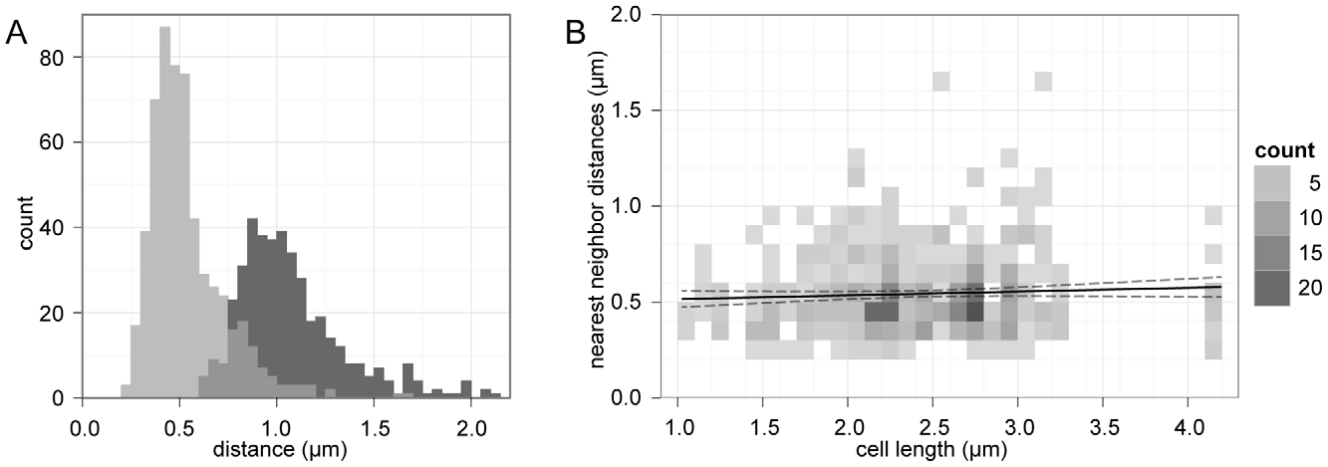
Nearest neighbor distributions of GFP-VirB8 foci. A, GFP-VirB8 foci distances from 76 cells shown as two superimposed histograms of peak to peak distances between nearest neighbor peaks (light grey) and next nearest neighbors (dark grey), with overlap in grey. Bin size is 0.05 µm. B, heat map of VirB8 nearest neighbor distances versus cell length. A linear model fit (black line) with 95% confidence intervals (dashed lines) show little correlation between cell length and foci spacing.

To assess whether this periodicity could be obtained by a random localization process, a precise set of nearest neighbor distances between foci was collected and analyzed. The distances between experimentally observed pairs of neighboring GFP-VirB8 foci were manually determined by measuring peak-to-peak distances of the recorded fluorescent profiles. When plotted on a histogram (Fig. 3A), we obtained two unimodal distributions with distinct peaks, demonstrating the median distances between nearest neighbor and next nearest neighbor VirB8 foci were 0.45 µm and 0.95 µm, respectively. The next nearest neighbor distribution peaks at twice the distance and with twice the width of the nearest neighbor distribution, indicating there is a consistent spatial separation between pairs and triplets of adjacent foci. The slight right-skew of these distributions could be explained if foci were stochastically absent along portions of a scaffold (compare ‘X’ and ‘2X’, Fig. 1B); this would result in occasional nearest neighbor measurements that span multiple periods.

Notably, the measured nearest neighbor distances and Fourier period closely agree with the simple average nearest neighbor distance (0.48 µm) that would be obtained if the 717 counted foci were distributed evenly over the total length of both sides of the measured cells (348 µm). This further demonstrates that the distribution of VirB8 foci in the cells was not biased towards any particular subcellular region or pole. For instance, if foci were found only towards one half of the cell, then these metrics would no longer coincide; the number of foci would be halved and the average distance between foci distributed over the entire cell length would be twice the average nearest neighbor distance. Furthermore, cell length did not correlate with VirB8 foci distances (Fig. 3B), indicating that cell size and cell cycle were not factors affecting foci spacing.

**Figure 4:**
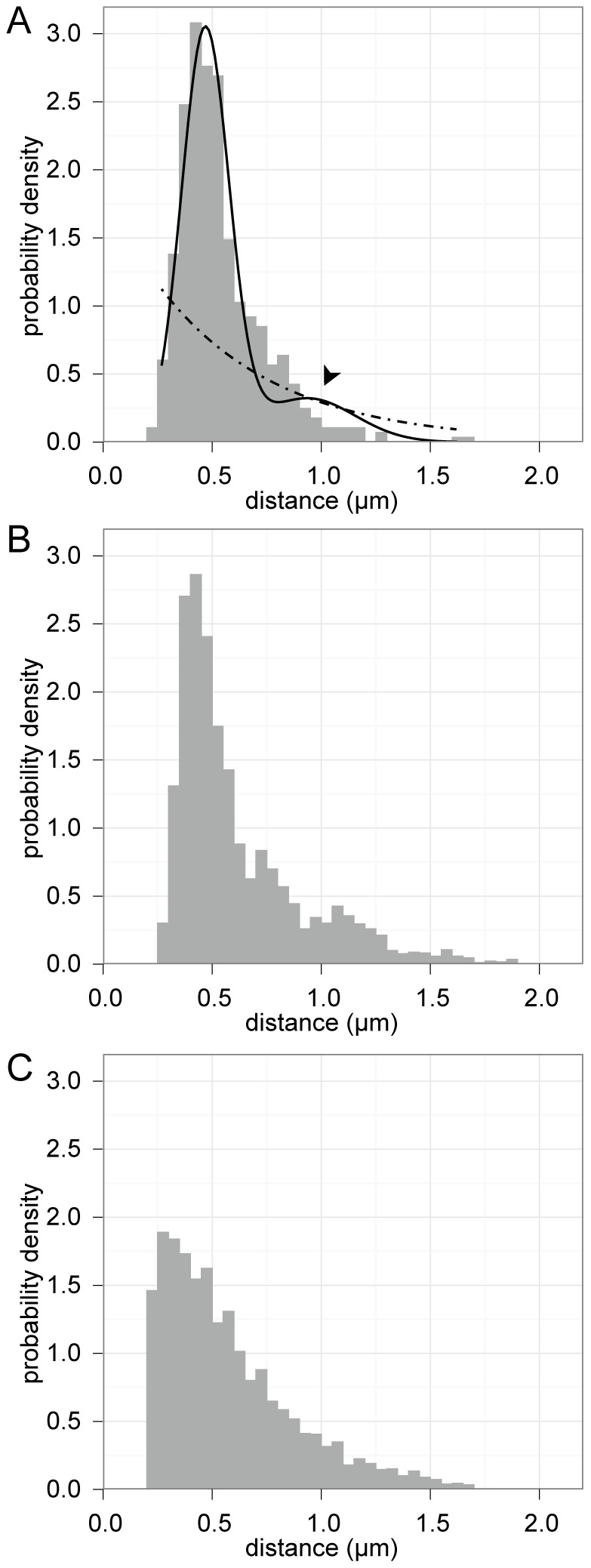
Maximum likelihood modeling and Monte Carlo simulations support periodic placement. A, density histogram of nearest neighbor distances for VirB8 overlayed with maximum likelihood best-fit models of periodic (—) and random (- • -) placement. Arrowhead indicates secondary peak of periodic model. B and C, density histograms of nearest neighbor distances gathered from Monte Carlo simulations of periodic helical (B) and random (C) placement.

To quantitatively evaluate if randomly distributed foci could produce the observed results, the experimental VirB8 nearest neighbor distribution (grey histogram, Fig. 4A) was fit with hypothetical distributions that would arise from either random (dashed line, Fig. 4A) or periodic (solid line, figure 4A) foci placement. A uniform random distribution of points on a line produces an exponential distribution of nearest neighbor distances (see Materials and Methods for derivation, and Fig. 4C for example); therefore an exponential distribution was used to model nearest neighbor measurements between foci along the edges of cells with randomly placed foci (dashed line, Fig. 4A). Periodic localization of foci was modeled as a Gaussian distribution with a mean (period) of μ and standard deviation σ (solid line, Fig. 4A). To account for the potential that nearest neighbor distances might occasionally span multiple periods, we introduced a small probability that pair distances were instead drawn from the next-nearest neighbor distribution (Gaussian with a mean of 2 µ and standard deviation 2σ). This resulted in a smaller secondary peak at twice the period of the primary distribution (arrowhead, Fig. 4A). The random and periodic models were then fit to the experimental VirB8 nearest neighbor distribution using maximum likelihood estimation; the resulting periodic model closely follows the observed distribution, while the random localization model fits the data poorly (Fig. 4A).

These model fits were next verified via Monte Carlo simulations of random and periodic foci localization. For each simulation, equal numbers of foci were repeatedly modeled on 3D cells in either a random or periodic helical pattern, then nearest neighbor distances along each edge were gathered in consideration of the resolution (∼200 nm) expected for deconvolved fluorescence microscopy. The Monte Carlo simulations (Fig. 4B and C) produced results mirroring the maximum likelihood model fits (Fig. 4A).

Finally, to evaluate the relative likelihoods of the periodic and random models, the Akaike information criterion (AIC) was used. The AIC allows for a quantitative comparison between two or more models with varying degrees of complexity by utilizing maximum likelihood estimates in conjunction with penalties for model complexity [34]. With this approach, the periodic model could be appropriately penalized for utilizing more parameters than the random model, thus ensuring that the better fit of the periodic model was not simply the result of over fitting the data. Given the data observed, the difference in AIC score was 841 in favor of the periodic localization model, indicating that random placement is extremely unlikely relative to the periodic model (E-value≪10^−100^). Thus, both visually and quantitatively, a model of periodically spaced foci better fit the measured distribution of VirB complexes than a null model of random spacing.

## Discussion

The localization of *virB* T4SS foci was assessed by Fourier analysis, nearest neighbor distributions, Monte Carlo cell simulations, maximum likelihood model fits and Akaike information criteria testing. Each analysis clearly indicated that the T4SS is not localized by a random process. Together, the data provide compelling evidence that VirB complexes are positioned periodically across the surface of *A. tumefaciens* cells. The data further demonstrate that the VirB complexes are distributed over the entire length of cells without bias towards any particular subcellular region, validating previous observations [18], [23].

It is not yet clear how the T4SS attains a periodic localization in *A. tumefaciens*, although one obvious possibility is that the T4SS complexes interact directly with a helical scaffold. A number of bacterial proteins have been reported to localize helically, most prominently the cytoskeletal proteins such as FtsZ and MinD [28]. Surprisingly, even some metabolic proteins such as CTP synthase and YvcK have been observed forming filaments or helices, respectively [35], [36]. Such proteins could serve directly as a scaffold guiding a helical, and therefore periodic, localization of T4SS complexes throughout the cell. Alternatively, structures consisting of concentric rings or multiple lateral filaments [37], [38] could also serve as scaffolds for generating regularly spaced T4SS complexes.

However, a protein scaffold is not the only way to acquire a periodic localization pattern. For instance, lipid rafts were recently implicated in heterologous protein distributions in *Bacillus subtilis* [39], and could potentially play a role in organizing the T4SS. Although not studied in *A. tumefaciens*, anionic lipids have also been reported to form regular bands or helices in *B. subtilis*, often also in conjunction with the Sec secretion system [25], [40], [41]. Many proteins, such as FtsA [42], SecA [25], [43], MinD [44], and MreB [45] interact directly with anionic lipids through positively charged amphipathic α-helical domains. Several of the VirB proteins, particularly the ATPase VirB11, contain putative amphipathic helices that could interact with anionic lipids to help direct the T4SS to anionic lipid domains.

In a closely related alternate mechanism, the T4SS complexes could also be localized by the Sec secretion system. Of the eleven T4SS components, eight (VirB1–3, VirB5, VirB7–10) are putative Sec secretion substrates (like many autotransporter proteins of Type V secretion [46], several of the VirB proteins of *A. tumefaciens* possess extended N-terminal Sec signal peptides that are not readily identified by prediction tools such as SignalP). With most of the T4SS components directed through the Sec secretion system, VirB complexes could be assembled in close proximity to Sec channels. Given the association of the Sec system with anionic lipids, and in conjunction with potential amphipathic helices within the VirB proteins, a regular banded or helical localization pattern could be imparted to the final assembled T4SS complexes.

One final possibility instead relies on disruptions of the peptidoglycan layer to facilitate assembly of the T4SS. The 20 nm T4SS core complex [12] likely requires significant remodeling of local peptidoglycan to successfully assemble in the cell envelope. Although VirB1 does function as a lytic transglycosylase, it may not sufficiently disrupt the peptidoglycan on its own. Localization of the VirB complexes near sites of peptidoglycan synthesis or modification would facilitate both insertion of the complex and the subsequently required repairs to the peptidoglycan layer.

Once a characteristic spacing of T4SS complexes is established, it would need to be maintained consistently through the course of cell growth. In bacterial species such as *E. coli*, a periodic pattern of T4SS complex assembly might become distorted over time as lateral peptidoglycan synthesis during cell elongation introduces new peptidoglycan between T4SS complexes. Interestingly, no correlation was found between cell length and foci spacing in *A. tumefaciens*, so cell growth does not appear to have a major role in determining or modifying foci placement. In further support, recent evidence indicates that *A. tumefaciens* undergoes primarily unipolar growth during cell elongation [47], [48], and therefore spatial arrangements of foci over the length of the cell would remain relatively undisturbed through successive rounds of cell division. Coupled with the dramatically slowed growth of *A. tumefaciens* under *vir-*induced conditions, periodically organized T4SS complexes most likely exist well through the duration of the infection process.

Compared to random localization, an organized distribution of the *virB* T4SS complexes could provide a variety of benefits to the bacterial cell. Fewer complexes would be required to ensure coverage of the bacterial circumference, thereby allowing cells to conserve resources spent on T4SS assembly. Furthermore, with VirB complexes evenly distributed across the cell surface and oriented in all directions, *A. tumefaciens* cells would be more likely to successfully contact a host cell and maintain stable lateral attachment for DNA and protein transport. Finally, a periodic T4SS distribution would help avoid excessive localized cell envelope stress that might occur due to complex clustering, a potential hazard given that each secretion channel is at least 20 nm in diameter [12] and spans both cell membranes and the peptidoglycan layer.

Further research will be required to explore the potential mechanisms and importance of periodic T4SS localization. Ultimately, such studies will improve our understanding of the *virB*-dependent pathogenesis of *A. tumefaciens*, and more generally, of the strategies bacterial cells employ to arrange the T4SS and other large cellular components.

## Materials and Methods

### Strains and Growth Conditions

Wild-type *A. tumefaciens* strain C58 containing nopaline pTiC58 was transformed with plasmid pJZ041 containing GFP-VirB8 under control of the *vir* promoter, as described [23]. Transformed cells were grown with 300 µM streptomycin and 100 µM spectinomycin under all conditions. To induce the *vir* system, an overnight culture was grown in LB at 28°C, then diluted to an OD_600_ of 0.1 in pH 5.5 minimal AB media and grown for 5 h at 19°C [11]. Cultures were plated on AB agar plates supplemented with 200 µM acetosyringone (AS) and incubated for 2 days at 19°C.

### Fluorescence imaging and measurements

*vir*-induced cells were resuspended in AB media to an OD_600_ of 5, and 5 µl were placed between a slide and coverslip. Stacks of optical sections were taken with an Applied Precision Deltavision Spectris DV4 deconvolution microscope and deconvolved using Huygens Pro (Scientific Volume Imaging) as described [23]. To acquire nearest neighbor distances and fluorescent profiles, deconvolved z-stacks were flattened into average intensity z-projections. To acquire fluorescent profiles, cell edges were selected manually with consideration of the corresponding brightfield images then measured over a 2-pixel thick averaged line using the profile tool in ImageJ [49]. All distinguishable individual cells in the field of view were included for analysis.

### Checking for periodicity using Fourier transforms

The centers of foci were determined automatically from fluorescent profiles as follows. The fluorescence intensity values of the profiles yielded one dimensional intensity-distance data: *I* = *I*(*x*). Foci centers were located to sub-pixel precision by interpolating a parabola to the three brightest data points at each putative peak of the fluorescent profiles. Since variations in foci size and brightness can obscure the periodicity of foci placements, the intensity curve for each focus was standardized by replacing its peak by a standard curve *I*(*x*,x_*_)∼exp(−(*x*−x_*_)^2^/2*w* ^2^), where x_*_ is the location of the peak, and the width of each standard curve was taken to be *w*≈Δ*x*, where Δ*x* is the spacing of measurements. For these standardized intensity curves, Fourier transforms could be calculated exactly *Î*(*k*,x_*_)∼exp(−π*w* ^2^ *k* ^2^−ikx_*_). To normalize and aggregate Fourier transforms from different intensity curves, the standardized intensity curves were subsampled on a regular grid: *k* was restricted to take discrete values {*k*_n_} = {2π*n*/*L : n = …*,−1,0,1, *…*}. Periodicity at any of the wave numbers *k_n_* leads to a large value of the |*Î*(*k_n_*)|; otherwise, the different phases of the contributions from different intensity peaks, *Î*(*k*,x_*_), tend to cause their Fourier transforms to cancel. To search for a signal of periodicity across many different cells, a histogram of relative frequencies was constructed by binning and averaging the discrete data |*Î*(*k_n_*)| across cells.

### Hypothesis testing

The experimentally measured inter-foci separations were compared with the predicted distribution of inter-foci separations assuming that foci were spaced at random around the cell. What is the distribution of foci separations under this null hypothesis? Suppose we know that there are *N* foci distributed along a cell of length *L*. Then the locations {*X_i_*} of the foci can be treated as uniform random variables, i.e. *X_i_*∼*U*(0,*L*). Each *X_i_* therefore has the same probability density function *p_i_*(*x*) = 1/*L*, 0<*x*<*L*. Suppose we are interested in the spacing *D_i_* between *X_i_* and the closest member of the set {*X_j_*: *X_j_>X_i_*, *j*≠*i*}. Given that *X_i_* = *x* (say) the likelihood that this spacing exceeds *d* is


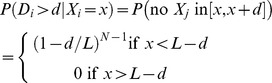


In the first case, (N−1) random variables {*X_j_*: *j*≠*i*} need to be chosen to fall outside of the interval, each, independently, with probability 1−*d*/*L*. To obtain the distribution of *D_i_* without conditioning on the location of *X_i_* we appeal to the law of total probability:


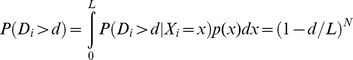


From this calculation we can calculate the probability density function for the separation of foci:


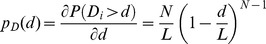


noting that the distribution is the same for all foci. In most cases, *d* is much smaller than *L*. We therefore consider the limit where *N* is allowed to tend to infinity, while keeping the mean inter-foci spacing constant λ = *L*/*N*. Then:


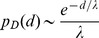


i.e. the inter-foci separations are exponentially distributed under the null hypothesis.

To calculate the likelihood that the measured distribution of foci separations arises from random placement, the Akaike Information Criterion (AIC) was calculated assuming (i) the null hypothesis and (ii) that foci had a preferred separation d_*_, modeled statistically, by a (Gaussian) *N*(*μ*,*σ*) distribution. Based on the observed data, two modifications were made to these distributions (i) to avoid modeling inter-foci separations below the observable limits of resolution, the null hypothesis distribution was modified by imposing a cut-off length scale *d′*, (ii) because variations in foci placement on a periodic substructure can lead to segments of this scaffold to occasionally lack foci (over the particular contours where the fluorescent profiles were collected), we allowed inter-foci separations to include pairs of foci from next nearest neighbor periods, along with pairs from neighboring periods. Accordingly, the null and alternate hypotheses were modeled statistically by distributions:


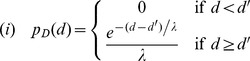



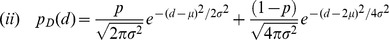


Here the parameter *p* represents the probability that a pair of foci is drawn from neighboring (rather than next-to-neighboring) periods. The parameters *d′*, *λ*, *σ*, *μ*, *p* were estimated by non-linear fitting to the observed data using the Matlab built-in function fminsearch (Mathworks, Waltham MA).

### Monte Carlo simulations

For each of the periodic and random foci simulations, 1000 *A. tumefaciens* cells were modeled as 3D cylinders 2 µm in length and 0.6 µm in diameter. The pitch and variability of the helical model, and the number of foci modeled were estimated from the VirB8 nearest neighbor data. Based on the VirB8 data, these models should include approximately 4 foci along each edge. However, it was estimated that foci as far as 150 nm from the cell edge (in a 2D projection) would be included in the fluorescent profiles measured over a 2-pixel (∼90 nm) thick averaged line, due to the inherent resolution limits of roughly 200 nm for fluorescence deconvolution microscopy [50]. Consequently, the 4 modeled foci on each cell edge would originate from ∼1/3 of the cell surface, indicating there should be 12 foci total for a cell 2 µm in length. Therefore, 12 foci were placed on the surface of each modeled cell, either at random positions along the path of a helix with a period of 0.50 µm±0.15 µm, or entirely at random. Nearest neighbor distances were then gathered between foci falling within 150 nm of each side of a 2D projection of each cell. To account for limits of resolution, the positions of foci falling closer than 200 nm were averaged together.

## Supporting Information

Figure S1 **Monte Carlo simulations of random and periodic placement.** Examples of (A) helical and (B) random localization of 16 foci simulated in 3-D on model *A. tumefaciens* cells 2.0 um in length. These types of simulations were used to collect data for Figure 4B, using only the foci along the sides of the cells.
(TIF)

Video S1 **3-D Rotations of representative** ***A. tumefaciens*** **cells expressing GFP-VirB8.** Maximum intensity projection rotations were generated from deconvolved stacks using ImageJ.
(MP4)

Video S2 **3-D Rotations of representative** ***A. tumefaciens*** **cells expressing GFP-VirB8.** Maximum intensity projection rotations were generated from deconvolved stacks using ImageJ.
(MP4)
