# Supplementary figures and images for: Quantitative Image Analysis and Modeling Indicate the Agrobacterium tumefaciens Type IV Secretion System Is Organized in a Periodic Pattern of Foci
Source: PLoS One. 2012 Jul 30;7(7):e42219. doi: 10.1371/journal.pone.0042219 (PMC3408489; doi:10.1371/journal.pone.0042219)

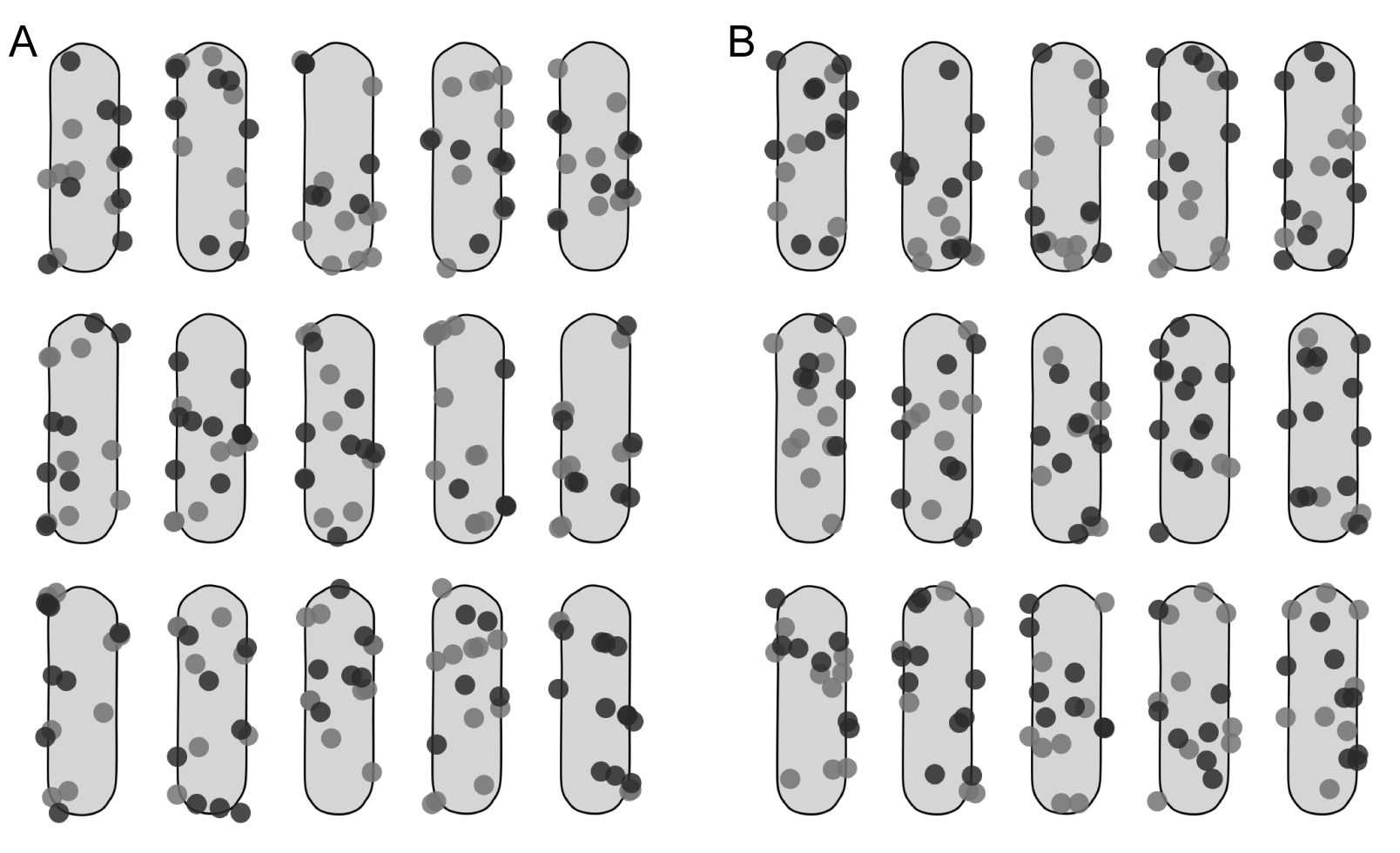

Supplement: Figure S1 — Monte Carlo simulations of random and periodic placement. Examples of (A) helical and (B) random localization of 16 foci simulated in 3-D on model A. tumefaciens cells 2.0 um in length. These types of simulations were used to collect data for Figure 4B, using only the foci along the sides of the cells. (TIF) [file pone.0042219.s001.tif]
